# Maternal intake of methyl-group donors affects DNA methylation of metabolic genes in infants

**DOI:** 10.1186/s13148-017-0321-y

**Published:** 2017-02-07

**Authors:** Sara Pauwels, Manosij Ghosh, Radu Corneliu Duca, Bram Bekaert, Kathleen Freson, Inge Huybrechts, Sabine A. S. Langie, Gudrun Koppen, Roland Devlieger, Lode Godderis

**Affiliations:** 1 Department of Public Health and Primary Care, Environment and Health, KU Leuven - University of Leuven, Kapucijnenvoer 35 blok D box 7001, 3000 Leuven, Belgium; 2 Flemish Institute of Technological Research (VITO), Unit Environmental Risk and Health, Boeretang 200, 2400 Mol, Belgium; 3 Department of Imaging & Pathology, KU Leuven - University of Leuven, 3000 Leuven, Belgium; 4 University Hospitals Leuven; Department of Forensic Medicine; Laboratory of Forensic Genetics and Molecular Archeology, KU Leuven - University of Leuven, 3000 Leuven, Belgium; 5 Center for Molecular and Vascular Biology, KU Leuven - University of Leuven, UZ Herestraat 49 - box 911, 3000 Leuven, Belgium; 6 International Agency for Research on Cancer, 150 Cours Albert Thomas, 69372 Lyon, CEDEX 08, France; 7 Faculty of Sciences, Hasselt University, 3590 Diepenbeek, Belgium; 8 Department of Development and Regeneration, KU Leuven - University of Leuven, 3000 Leuven, Belgium; 9 Department of Obstetrics and Gynecology, University Hospitals of Leuven, 3000 Leuven, Belgium; 10 IDEWE, External Service for Prevention and Protection at Work, Interleuvenlaan 58, 3001 Heverlee, Belgium

**Keywords:** Methyl-group donors, DNA methylation, LEP, IGF2 DMR, RXRA, DNMT1, Lactation, Pregnancy

## Abstract

**Background:**

Maternal nutrition during pregnancy and infant nutrition in the early postnatal period (lactation) are critically involved in the development and health of the newborn infant. The Maternal Nutrition and Offspring’s Epigenome (MANOE) study was set up to assess the effect of maternal methyl-group donor intake (choline, betaine, folate, methionine) on infant DNA methylation. Maternal intake of dietary methyl-group donors was assessed using a food-frequency questionnaire (FFQ). Before and during pregnancy, we evaluated maternal methyl-group donor intake through diet and supplementation (folic acid) in relation to gene-specific (*IGF2* DMR, *DNMT1*, *LEP*, *RXRA*) buccal epithelial cell DNA methylation in 6 months old infants (*n* = 114) via pyrosequencing. In the early postnatal period, we determined the effect of maternal choline intake during lactation (in mothers who breast-fed for at least 3 months) on gene-specific buccal DNA methylation (*n* = 65).

**Results:**

Maternal dietary and supplemental intake of methyl-group donors (folate, betaine, folic acid), only in the periconception period, was associated with buccal cell DNA methylation in genes related to growth (*IGF2* DMR), metabolism (*RXRA*), and appetite control (*LEP*). A negative association was found between maternal folate and folic acid intake before pregnancy and infant *LEP* (slope = −1.233, 95% CI −2.342; −0.125, *p* = 0.0298) and *IGF2* DMR methylation (slope = −0.706, 95% CI −1.242; −0.107, *p* = 0.0101), respectively. Positive associations were observed for maternal betaine (slope = 0.875, 95% CI 0.118; 1.633, *p* = 0.0241) and folate (slope = 0.685, 95% CI 0.245; 1.125, *p* = 0.0027) intake before pregnancy and *RXRA* methylation. Buccal *DNMT1* methylation in the infant was negatively associated with maternal methyl-group donor intake in the first and second trimester of pregnancy and negatively in the third trimester. We found no clear association between maternal choline intake during lactation and buccal infant DNA methylation.

**Conclusions:**

This study suggests that maternal dietary and supplemental intake of methyl-group donors, especially in the periconception period, can influence infant’s buccal DNA methylation in genes related to metabolism, growth, appetite regulation, and maintenance of DNA methylation reactions.

## Background

During pregnancy, environmental exposures can influence the development of the offspring and increase the risk for metabolic diseases, like obesity, later in life. One maternal factor that has consistently been shown to influence later phenotype is maternal nutrition [1]. This has been most clearly shown in studies of the Dutch Hunger Winter (1944–1945), a 5-month period of extreme food shortage in the Netherlands at the end of World War II. Long-term follow-up studies from this cohort found that exposure to famine in early gestation was associated with low birth weight and increased risk of obesity in adulthood, whereas, exposure in late gestation showed decreased glucose tolerance [2]. Studies from this cohort indicate that there are different windows of susceptibility during pregnancy (embryogenesis, organogenesis, and tissue differentiation) where maternal nutrition can influence offspring’s health [3]. In addition, the early postnatal period is another critical period in which nutrition can program the infant. Several physiological and metabolic mechanisms are not fully mature at birth and continue to develop in the immediate postnatal period [4].

One of the underlying mechanisms responsible for metabolic programming is epigenetic modifications, such as DNA methylation [5]. The process of methylation and demethylation is a natural process allowing the cell to grow and differentiate. Shortly after fertilization, DNA methylation marks on the maternal and paternal genome are globally demethylated, which is followed by de novo methylation just before implantation. This is a critical window of fetal development during pregnancy where dietary factors can influence the fetal methylome [6]. Methyl-group donors derived from food (choline, betaine, folate, and methionine) and supplements (folic acid), which contains a methyl-group (CH_3_), enter the one-carbon (I-C) metabolism at different sites and are, in the end, converted to the universal methyl-group donor S-adenosylmethionine (SAM). SAM will donate a methyl-group for the methylation of the DNA [7]. Choline plays a role in the structural integrity of cell membranes, in the lipid-cholesterol transport and metabolism, and in normal brain development (precursor of acetylcholine) [8, 9]. Betaine is essential in the preimplantation embryo, in which it may play a role as an osmolyte, and for correct neural tube formation [10]. The intake of folate or vitamin B_9_ (400 μg per day) is recommended during pregnancy to prevent neural tube defects, placental abruption, preterm birth, and low birth weight [11, 12]. Methionine is an indispensable amino acid required for protein synthesis. Diets with an inappropriate balance of methionine can adversely affect fetal development [13].

Many animal studies examined the effect of maternal methyl-group-supplemented diets on offspring epigenome, health, and longevity. A classic example is the agouti viable yellow mouse, which has a yellow coat color, is obese, hyperinsulinemic, and is more susceptible to cancer. Maternal dietary supplementation with methyl-group donors shifts the coat color of the offspring towards the brown pseudoagouti phenotype and lowers the disease risk [14]. Animal models have confirmed the biological possibility of fetal programming in response to maternal methyl-group donor supplementation and make it reasonable to think that similar processes could happen in humans. Until now, some studies in humans have shown that maternal methyl-group donor intake can influence offspring methylation. For example, Increased *IGF2* methylation and decreased *PEG3* and LINE-1 methylation were observed in cord blood with increased folic acid supplement consumption after 12 weeks of pregnancy [15]. However, the long-term effects on offspring health remain unknown in humans.

Methyl-groups are transferred from SAM to the DNA by DNA methyltransferases (*DNMTs*). *DNMT1* is responsible for maintaining DNA methylation patterns through mitosis [16]. *DNMT3A* and *DNMT3B* are responsible for the establishment of new or “de novo” DNA methylation patterns during early embryogenesis, which is a vulnerable period where nutritional insults can disrupt the correct establishment of epigenetic marks. According to Heijmans et al. [5], a decrease of 5.2% in insulin-like growth factor 2 (*IGF2*) differentially methylated region (DMR) whole blood DNA methylation was observed in 60 adults exposed to periconception famine compared to same-sex siblings who were not exposed. This association was only seen when there was an exposure in early gestation, not in mid or late gestation. *IGF2* is a maternally imprinted gene that is important for fetal growth and development. The *IGF2* DMR is only methylated on the maternal allele, so this region might be vulnerable to nutritional exposures in the pre- and periconception period [17]. Another study from the Dutch Hunger Winter found a significant increase in leptin (*LEP*) whole blood DNA methylation of men exposed to famine in early and late gestation. These results suggest that environmentally induced DNA methylation changes may not be limited to the periconception period (period starting 14 weeks before conception until 10 weeks postconception) [18] but it appears to extend to the whole prenatal period [19]. Leptin is a hormone, produced by adipose tissue, which is implicated in appetite control (inhibits food intake) and fat metabolism. *LEP* promoter methylation differences can influence *LEP* expression [20]. Godfrey et al. [21] observed that lower maternal carbohydrate intake in early pregnancy was associated with higher methylation of the retinoid X receptor-α (*RXRA*) gene in umbilical cord blood. In addition, the authors found that greater methylation levels in *RXRA* were more strongly correlated with greater adiposity (fat mass and percentage fat mass) in later childhood (9 years old) in two independent cohorts. *RXRA* is known to have beneficial effects on insulin sensitivity, adipogenesis, and fat metabolism, through its binding to the transcription factor peroxisome proliferator-activated receptor (*PPAR*) [22].

Early postnatal life has shown to be another critical window for metabolic programming in which nutrition can induce epigenetic changes in the infant. In the early postnatal period, newborns are either breast-fed or formula-fed. The ideal nutrient composition of breast milk and the peculiar feeding behavior associated with breastfeeding seem to have a protective effect against the development of obesity later in life. However, the different epigenetic mechanisms involved remain unclear [23]. One study found that the duration of breastfeeding was negatively associated with *LEP* whole blood methylation in 17 months old children. It was hypothesized that the breast milk content contributes to programming of the neuroendocrine system by changing *LEP* methylation. The decrease in *LEP* methylation could be one of the mechanisms by which breastfeeding contributes to protection against childhood obesity [20]. Some human studies hypothesize that specific breast milk components could possibly induce epigenetic changes and influence the child’s health outcome. For example, the high cholesterol content of breast milk may reduce endogenous cholesterol synthesis, probably by down-regulation of hepatic hydroxymethyl glutaryl coenzyme A (HMGCoA) reductase through epigenetic mechanisms [23]. Consequently, it is important to investigate the effect of maternal dietary choline intake during lactation on infant DNA methylation levels. Hence, the methyl-group donor choline can influence choline breast milk composition and infant choline status. Folate breast milk concentration on the other hand is maintained even when the mother is folate deficient and is unaffected by maternal folic acid supplementation [24]. Methionine is present in breast milk in low concentrations. Amino acid composition of breast milk can be influenced by lactation stage but not by maternal dietary protein intake [25, 26].

In this study, we investigated the effect of maternal dietary methyl-group donor intake (choline, betaine, folate, and methionine) and supplemental intake (folic acid) before and during each trimester of pregnancy on gene-specific methylation (*DNMT1*, *IGF2* DMR, *RXRA*, and *LEP)* in buccal epithelial cells of 6 months old infants. Buccal swabs were chosen to collect DNA because the samples are easy to collect and it is a non-invasive technique, which is important to consider when taken DNA samples from infants. Buccal samples mainly exist of exfoliated (dead) epithelial cells but have a more homogenous cell population compared to blood samples [27]. Next, we determined the effect of maternal choline intake during lactation, in mothers who breast-fed for at least 3 months, on gene-specific buccal DNA methylation. In the gene-specific DNA methylation analysis, we included *DNMT 1*, *IGF2* DMR, *RXRA*, and *LEP*.

## Methods

### Study subjects

**Fig. 1:**
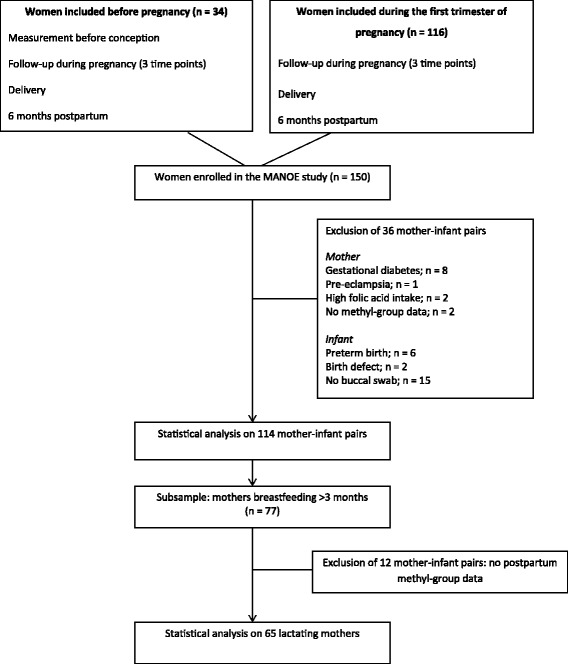
Flowchart of mother–infant pairs enrolled in the MANOE study and included in the statistical analysis

We studied participants enrolled in the MANOE (Maternal Nutrition and Offspring’s Epigenome) study, an ongoing prospective, observational cohort study initiated in April 2012. Healthy Caucasian women who desired to become pregnant or who were in the first trimester of pregnancy were recruited at the Department of Obstetrics and Gynecology of the University Hospitals Leuven (Belgium). We enrolled 150 women (34 women before pregnancy and 116 in the first trimester of pregnancy) between April 2012 and January 2015. The last delivery of the cohort took place in September 2015 and the last 6 months postpartum (PP) visit in March 2016. Exclusion criteria were the following: non-Caucasian women, multiple pregnancies (twins, triplets, etc.), and infertility treatment. Of the 150 enrolled women, 36 mother–infant pairs were excluded from analysis due to missing nutritional data (*n* = 2), missing buccal swab samples (*n* = 15), development of pregnancy complications (gestational diabetes (*n* = 8) and preeclampsia (*n* = 1)), preterm delivery (*n* = 6), extreme high intake of folic acid (4 mg/day) (*n* = 2), and birth defects (*n* = 2). This gives us a total of 114 mother–infant pairs for statistical analysis. Further statistical analysis was performed on a subsample of 65 lactating mother–infant pairs. A flowchart of the mother–infant pairs enrolled in the MANOE study and included in the statistical analysis is presented in Fig. 1. The recruitment process has been described in more detail in a previous study [28].

This study was conducted according to the guidelines laid down in the Declaration of Helsinki and all procedures involving human subjects were approved by the UZ Leuven-Committee for Medical Ethics (reference number: ML7975). Written informed consent was obtained from all subjects.

### Maternal and neonatal measurements

All 114 women were followed up during pregnancy at their scheduled ultrasounds (11–13 weeks, 18–22 weeks, and 30–34 weeks of gestation), 6 weeks, and 6 months PP. From the women recruited before pregnancy (*n* = 34), extra measurements were taken before conception. To assess maternal intake of dietary methyl-group donors (methionine, folate, betaine, and choline) before pregnancy, during each trimester of pregnancy, and PP, a food-frequency questionnaire (FFQ) was developed, validated [29, 30], and implemented in the MANOE study. The FFQ contains 51 food items and women were asked to indicate their answers in a list of frequencies and portion sizes to calculate the usual daily intake of the four methyl-group donors (mg or μg/day). Twenty-one FFQs were obtained before pregnancy, 94 FFQs at 11–13 weeks, 85 FFQs at 18–22 weeks, 82 FFQs at 30–34 weeks of pregnancy, 79 FFQs 6–8 weeks PP, and 60 FFQs 6 months PP. To assess the intake of methyl-group donors through supplement use, questions were asked about the use of nutritional supplements (frequency, brand/type, dosage) before, during each trimester of pregnancy, and PP. Only the intake of folic acid (synthetic form of folate) was registered, since there was no report on the supplemental intake of methionine, betaine, and choline. Furthermore, using a combination of questionnaires and interviews, we collected information about a range of socio-demographic factors, life style habits, and physical activity. Information on mothers’ smoking status before and during pregnancy was obtained at each consultation. Questions were asked about smoking before and in each trimester of pregnancy and the number of cigarettes smoked on average per day. From these data, a dichotomous variable for maternal smoking before and during pregnancy was derived (did not smoke/smoked). Height and prepregnancy weight were used to calculate the prepregnancy body mass index (BMI, kg/m^2^).

Six months after birth, data on breastfeeding was derived and scores were given ranging from 0–4 (0 = formula feeding; 1 = <1 month of breastfeeding; 2 = 1–3 months of breastfeeding; 3 = 3–6 months of breastfeeding; 4 = >6 months of breastfeeding). We measured infant weight and length at the 6 months PP visit. Maternal and neonatal measurements have been described in more detail in a previous study [28].

### Sample collection and DNA extraction

A Cytobrush plus Medscan® was used to brush against the inner cheeks of the infant. The brush handle was cut off and put inside a 15-mL Falcon tube with PBS and stored immediately at −20 °C, until DNA extraction. DNA extraction from cytobrush was performed using the QIAamp DNA Blood Mini Kit (Qiagen Inc., Valencia, CA). The final elution volume obtained was 100 μL. The quantity and purity of DNA were determined by a NanoDrop spectrophotometer.

### Gene-specific DNA methylation measurements

#### Bisulfite conversion and PCR

Genomic DNA (200 ng) was bisulfite converted using the EZ-96 DNA Methylation-Gold™ Kit (#D5008, Zymo Research). Converted DNA was eluted with 30 μL of M-elution buffer. Subsequently, 1 μL of converted DNA was amplified by PCR in a total volume of 25 μL containing 0.2 μM of primers and 2× Qiagen PyroMark PCR Master Mix (#978703, Qiagen). Primers for *DNMT1*, *RXRA*, and *LEP* were ordered from Qiagen (#PM00075761, #PM00144431, #PM00129724 PyroMark CpG Assays). The analyzed sequences are part of the promoter region and lie within a CpG island. For the *RXRA* gene, the analyzed sequence also lies in a transcriptional regulatory site. Primer sequences for *IGF2* DMR were taken from the original paper. Imprinting of the *IGF2* gene is regulated by this differentially methylated region which is located upstream of the imprinted promoters of *IGF2* exon 3 [31].

PCR for *DNMT1*, *RXRA*, and *LEP* consisted of an initial hold at 95 °C for 15 min followed by 45 cycles of 30 s at 94 °C, 30 s at 54 °C, and 30 s at 72 °C. PCR amplification ended with a final extension step at 72 °C for 10 min. PCR for *IGF2* DMR consisted of an initial hold at 5 °C for 15 min followed by 5 cycles of 30 s at 94 °C, 30 s at 68 °C, and 30 s at 72 °C. This was followed by 50 cycles of 30 s at 94 °C, 30 s at 64 °C, and 30 s at 72 °C and ended with a final extension step at 72 °C for 10 min.

#### Pyrosequencing

In order to assess CpG methylation levels, 20 μL of biotinylated PCR product was immobilized to Streptavidin Sepharose High Performance beads (#17-5113-01, GE Healthcare) followed by annealing to 25 μL of 0.3 μM sequencing primer at 80 °C for 2 min with a subsequent 10 min cooling down period. Pyrosequencing was performed using Pyro Gold reagents (#970802, Qiagen) on the PyroMark Q24 instrument (Qiagen) following the manufacturer’s instructions. Pyrosequencing results were analyzed using the PyroMark analysis 2.0.7 software (Qiagen). Five CpGs were analyzed for *DNMT1*, three CpGs for *IGF2* DMR, four CpGs for *LEP*, and five CpGs for *RXRA*. Six samples were randomly selected for technical variation analysis.

### Statistical analysis

First, we assessed changes in the intake of maternal methyl-group donors during and after pregnancy using a multivariate regression model for longitudinal measurements with methyl-group donor intake as a response variable and time point as a factor (LSD post hoc test).

Next, we determined the effect of maternal methyl-group donor intake on gene-specific DNA methylation (*IGF2* DMR, *LEP*, *RXRA*, *DNMT1*) using linear mixed models. Linear mixed models were used with gene-specific DNA methylation as a response variable and methyl-group donor intake, CpG site, and their interaction as explanatory variables. Other covariates were included in the multivariable model to correct for possible confounding. Potential confounders were selected based on the association with infant DNA methylation and maternal nutrition: maternal age, maternal prepregnancy BMI, maternal smoking before and during each trimester of pregnancy (0 = did not smoke before and during pregnancy, 1 = smoked before and during pregnancy), gestational weight gain, and duration of breastfeeding (0 = formula feeding; 1 = <1 month of breastfeeding; 2 = 1–3 months of breastfeeding; 3 = 3–6 months of breastfeeding; 4 = > 6 months of breastfeeding). A random intercept was modeled to deal with the clustered nature of the data. Analyses were performed separately per time point (prepregnancy, 11–13 weeks pregnancy, 18–22 weeks pregnancy, 30–34 weeks pregnancy). First, the interaction between maternal methyl-group donor intake and CpG site was tested. A significant interaction test implies that the association between methyl-group donor intake and CpG methylation is different between the individual CpGs. In this case, results were reported per individual CpG. A non-significant interaction test indicates lack of evidence for a differential association between methyl-group donor intake and methylation at different CpGs. In this case, a main effect of methyl-group donor intake over the different CpGs was reported.

Next, an independent *t* test was performed on a subsample of lactating women (*n* = 65) to assess the effect of maternal choline intake during breastfeeding on buccal DNA methylation at 6 months. The mean maternal methyl-group donor intake during the 6 months after delivery was calculated using the two FFQs administrated during this period and using supplement information. Finally, we determined the effect of maternal choline intake during lactation on gene-specific DNA methylation (*IGF2* DMR, *LEP*, *RXRA*, *DNMT1*) using linear mixed models. Linear mixed models were used with gene-specific DNA methylation as a response variable and choline intake, CpG site, and their interaction as explanatory variables. Other covariates were included in the multivariable model to correct for possible confounding. Potential confounders were selected based on the association with infant DNA methylation and maternal nutrition: maternal age, maternal prepregnancy BMI, maternal smoking, and gestational weight gain. A random intercept was modeled to deal with the clustered nature of the data. Analyses were performed separately per time point (0–3 months postpartum, 3–6 months postpartum). First, the interaction between maternal choline intake and CpG site was tested. A significant interaction test implies that the association between choline intake and CpG methylation is different between the individual CpGs. In this case, results were reported per individual CpG. A non-significant interaction test indicates lack of evidence for a differential association between choline intake and methylation at different CpGs. In this case, a main effect of choline intake over the different CpGs was reported.

All tests were two-sided, a 5% significance level was assumed for all tests. Analyses have been performed using SAS software (version 9.4 of the SAS System for Windows).

## Results

Characteristics of the 114 mother–infant pairs included in the statistical analysis are presented in Table 1. The mean maternal age and standard deviation (SD) was 31 ± 3.7 years, mean prepregnancy BMI and SD was 23 ± 3.4 kg/m^2^, and the mean gestational weight gain and SD was 14.8 ± 4.1 kg. Only four women smoked before and during the first trimester of pregnancy. One woman continued smoking during the second and third trimester. The infants, 54 of which were girls (47.4%), had a mean weight and SD of 7875.4 ± 877.6 g, a mean length and SD of 67.9 ± 2.6 cm, and the mean age and SD was 6.3 ± 2.4 months. Only 7% of the women decided to exclusively use formula feeding, while the biggest group of women (39.5%) breastfed for more than 6 months.

**Table 1:** Maternal and infant characteristics (*n* = 114)

Characteristics	Mean (SD)	Range
Mother		
Maternal age (years)	31 (3.7)	25–41
Prepregnancy BMI (kg/m^2^)	23 (3.4)	17.9–33
Gestational weight gain (kg)	14.8 (4.1)	5.3–28.9
Infant		
Weight (g)	7875.4 (877.6)	6240–11,120
Length (cm)	67.9 (2.6)	62–76.5
Age (months)	6.3 (0.4)	4.6–7.2
	%	*N*
Maternal smoking (yes)		
Before pregnancy	3.5	4
First trimester	3.5	4
Second trimester	0.9	1
Third trimester	0.9	1
Gender		
Boy	52.6	60
Girl	47.4	54
Duration of breastfeeding		
0 months	7	8
<1 month	6.1	7
1–3 months	19.3	22
3–6 months	28.1	32
>6 months	39.5	45

Most of the women in the study had a methionine intake above the daily requirement of 10.4 mg/kg body weight per day [32]. Dietary methionine intake was significantly lower 6 months PP (1533.6 mg/day) than the intake in the third trimester (1659.3 mg/day, *p* = 0.043) of pregnancy and 6–8 weeks PP (1678.5 mg/day, *p* = 0.01). The dietary intake of folate, choline, and betaine was stable and did not change during pregnancy and in the PP period (Table 2). All women took a folic acid supplement in the first trimester of pregnancy to reach an uptake of 400 μg of folate per day. Remarkably, some continued taking the supplement throughout pregnancy and lactation, despite the recommendation of starting 4 weeks before conception until 12 weeks of pregnancy [33].

**Table 2:** Intake of maternal methyl-group donors through diet and supplements (folic acid) during pregnancy and in the postpartum (PP) period

Methyl-group donors	First trimester (10–13 weeks) Mean (SE) Range *N* = 94	Second trimester (18–22 weeks) Mean (SE) Range *N* = 85	Third trimester (30–34 weeks) Mean (SE) Range *N* = 82	6–8 weeks PP Mean (SE) Range *N* = 79	6 months PP Mean (SE) Range *N* = 60	*p*	Dietary guidelines
Methionine (mg) (mg/kg)	1644.4 (45.9) 792–2932 12.4–45.1	1608.4 (44.3) 746.1–2684.4 9.6–38	1659.3 (48.7) 789–2957 9.2–40.6	1678.5 (52) 786.4–3499.4 12.5–45.8	1562.4^a^ (43.5) 710.5–2562.8 11.7–35.1	0.047	Daily requirement 10.4 mg/kg body weight
Folate (μg)	272.1 (8.7) 131–531	263.2 (8.8) 98–519.8	279.8 (10.5) 112–619	264 (9) 97.4–520.7	263.7 (10.7) 98–564.4	0.17	Recommended intake Non-pregnant 200-300 Pregnant 400 Lactation 300
Folic acid (μg)	507.2^b^ (14.1) 171–1000	399.9 (23.9) 0–1000	391.3 (25.6) 0–1000	204.3^c^ (24.7) 0–800	68^c^ (14.9) 0–600	0.000	
Choline (mg)	274.4 (7.4) 137–451	268.1 (7.4) 137.3–469.2	280.3 (8.6) 130–552	278.2 (8.5) 128.3–547.4	268.4 (7.8) 115.3–435.5	0.26	Adequate intake Non-pregnant 400 Pregnant 450 Lactation 550
Betaine (mg)	162.6 (5.7) 63–342	169.2 (6.2) 52.6–354.3	173.2 (6.7) 68–320	170.2 (6.5) 80–349.3	162.6 (7.2) 34.5–326.2	0.53	/

*p* values were obtained using a multivariate regression model for longitudinal measurements

^a^Methionine intake 6 months PP was significantly lower than the intake in the third trimester of pregnancy and 6–8 weeks PP

^b^Folic acid intake was significantly higher in the first trimester of pregnancy compared to the other time points

^c^Folic acid intake in the PP period was significantly lower than the intake during pregnancy and within the PP period, folic acid intake 6 months PP was lower

The supplemental intake of folic acid on the other hand was highest in the first trimester of pregnancy (507.2 μg) and significantly different from the folic acid intake in the other four time points (*p* = 0.000). Postpartum, the intake of folic acid was significantly lower as compared to the intake during every trimester of pregnancy. Within the PP period, the folic acid intake at 6 months (68 μg/day) was significantly lower than the intake at 6–8 weeks (204.3 μg/day, *p* = 0.000). For choline, the adequate choline intake was 425 mg for non-pregnant women, 450 mg for pregnant women, and 550 mg for lactating women [34]. Most women had an average intake of about 300 mg choline per day, which lies below the adequate intake. For betaine, no guideline for dietary intake exists.

### Gestational methyl-group donor intake and infant buccal DNA methylation

We estimated the association of maternal methyl-group donor intake before pregnancy and during each trimester of pregnancy on infant (6 months old) gene-specific DNA methylation (*DNMT1*, *IGF2* DMR, *RXRA*, and *LEP*) in buccal epithelial cells. The statistically significant associations and trends between maternal methyl-group donor intake and buccal DNA methylation are presented in Table 3.

**Table 3:** Associations between maternal methyl-group donor intake (before and during pregnancy) and infant *DNMT1*, *IGF2* DMR, *RXRA*, and *LEP* methylation in buccal epithelial cells

Time point	Before pregnancy			First trimester			Second trimester	Third trimester	
	B (95% CI) *p* value *N* = 21			B (95% CI) *p* value *N* = 94			B (95% CI) *p* value *N* = 85	B (95% CI) *p* value *N* = 82	
Gene	*RXRA* All CpG sites^a^	*LEP* All CpG sites^a^	*IGF2* DMR All CpG sites^a^	*IGF2* DMR		*DNMT1* All CpG sites^a^	*DNMT1* All CpG sites^a^	*DNMT1*	
Nutrient									
				CpG1^b^	All CpG sites^a^			CpG1^b^	CpG3^b^
Betaine	*0.875* *(0.118; 1.633)* *0.0241*				2.341 (−0.138; 4.82) 0.0640				
Choline						−0.092 (−0.191; 0.008) 0.07		*0.156* *(0.029; 0.283)* *0.0166*	
Folate	*0.685* *(0.245; 1.125)* *0.0027*	*−1.233* *(−2.342; −0.125)* *0.0298*							*0.131* *(0.016; 0.246)* *0.0256*
Folic acid			*−0.706* *(−1.242; −0.107)* *0.0101*	1.013 (−0.095; 2.121) 0.0728		−0.033 (−0.072; 0.006) 0.0987	*−0.027* *(−0.051; −0.004)* *0.0204*		
Methionine						−0.013 (−0.029; 0.002) 0.0831			

β-Estimate is an absolute change in percentage of methylation; slope >(<) 0 means positive (negative) association

^a^When there was no evidence for a differential association between maternal methyl-group donor intake and DNA methylation at the different CpG locations, the main effect of maternal methyl-group donor intake over all CpG locations was reported

^b^When there was a significant interaction test, the association between maternal methyl-group donor intake and DNA methylation was different between CpG locations. In this case, the results were reported per CpG location

*CI* confidence interval

Before pregnancy, maternal betaine, folate, and folic acid intakes were associated with buccal epithelial methylation levels of *RXRA*, *LEP*, and *IGF2* DMR. A higher intake of folate and betaine was associated with higher *RXRA* methylation across all CpGs (for folate, 0.685% increase in *RXRA* methylation per 100 μg folate increase; 95% CI 0.245, 1.125; *p* = 0.027; for betaine, 0.875% increase in *RXRA* methylation per 100 mg betaine increase; 95% CI 0.118, 1.633; *p* = 0.0241). In addition, a higher intake of folate was associated with lower *LEP* methylation across all CpGs (−1.233% decrease in *LEP* methylation per 100 μg folate increase; 95% CI −2.342, −0.125; *p* = 0.0298). For folic acid, a higher intake before pregnancy was associated with lower *IGF2* DMR methylation across all CpGs (−0.706% decrease in *IGF2* DMR methylation per 100 μg folic acid increase; 95% CI −1.242, −0.107; *p* = 0.0101).

In the first trimester of pregnancy, only borderline significant results were found between maternal methyl-group donor intake and *IGF2* DMR and *DNMT1* methylation.

In the second trimester of pregnancy, a higher intake of folic acid was associated with lower *DNMT1* methylation across all CpGs (−0.027% decrease in *DNMT1* methylation per 100 μg folic acid increase; 95% CI −0.051, −0.004; *p* = 0.0204).

In the third trimester of pregnancy, a higher intake of choline and folate was associated with higher *DNMT1* CpG1 methylation (0.156% increase in *DNMT1* CpG1 methylation per 100 mg choline increase; 95% CI 0.029, 0.283; *p* = 0.0166) and higher *DNMT1* CpG3 methylation (0.131% increase in *DNMT1* CpG3 methylation per 100 μg folate increase; 95% CI 0.016, 0.246; *p* = 0.0256), respectively.

#### Choline intake of lactating women and infant methylation

**Fig. 2:**
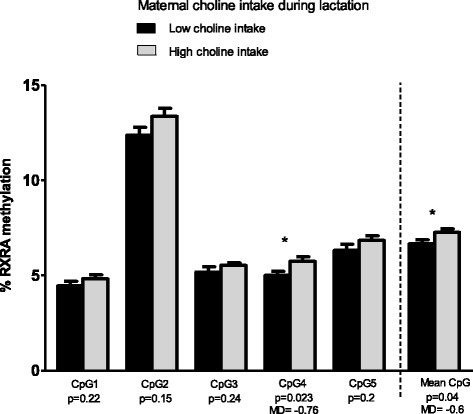
Buccal *RXRA* methylation by maternal dietary choline intake during lactation. The graphs represent the mean methylation values and standard error of the mean bars of 65 infants. The results are based on a low or high dietary maternal intake of choline during lactation. The overall *p* values and significant *p* values with mean differences (MD) are also shown

First, we found statistically significant differences in buccal *RXRA* methylation (CpG4 and mean CpG) between low and high (≥275.27 mg/day) maternal dietary intake of choline during lactation. The results are shown in Fig. 2. We observed significantly higher *RXRA* methylation percentages when the mother consumed a diet high in choline during lactation compared to a diet low in choline (for CpG4, 5.7 ± 1.4 vs. 5 ± 1.2%, *p* = 0.023, 95% CI −1.407, −1.083; for mean CpG, 7.3 ± 1.1 vs. 6.7 ± 1.2%, *p* = 0.04, 95% CI −1.180, −0.028).

Next, we estimated the association between choline intake of lactating women during two time points (0–3 months after pregnancy and 3–6 months after pregnancy) and infant gene-specific DNA methylation (*DNMT1*, *IGF2* DMR, *RXRA*, and *LEP*) in buccal epithelial cells. No significant association between maternal choline intake and infant DNA methylation levels was found (Table 4).

**Table 4:** Associations between maternal choline intake during lactation (0–3 months after pregnancy and 3–6 months after pregnancy) and infant DNMT1, IGF2 DMR, RXRA, and LEP methylation in buccal epithelial cells

Time point	0–3 months after pregnancy	3–6 months after pregnancy
Gene^a^	B (95% CI) *p* value *N* = 58	B (95% CI) *p* value *N* = 42
*DNMT1*	0.080 (−0.062; 0.223) 0.2675	−0.060 (−0.360; 0.240) 0.6925
*IGF2* DMR	−0.984 (−2.416; 0.449) 0.1764	0.479 (−2.157; 3.114) 0.7189
*RXRA*	0.291 (−0.057; 0.640) 0.1012	0.321 (−0.207; 0.850) 0.2319
*LEP*	−0.226 (−1.271; 0.818) 0.6695	0.057 (−1.713; 1.828) 0.9490

β-Estimate is an absolute change in percentage of methylation; slope >(<) 0 means positive (negative) association

^a^When there was no evidence for a differential association between maternal choline intake and DNA methylation at the different CpG locations, the main effect of maternal choline intake over all CpG locations was reported, which was the case for all genes

*CI* confidence interval

## Discussion

This research supports the hypothesis that maternal methyl-group donor intake before and during pregnancy could possibly induce epigenetic alterations in offspring genes related to metabolism and genes important to maintain DNA methylation patterns. We first studied the effect of maternal methyl-group donor intake (through diet and supplements) before and during each trimester of pregnancy on gene-specific DNA methylation (*IGF2* DMR, *LEP*, *RXRA*, *DNMT1*) in buccal epithelial cells of 6 months old infants. We observed that maternal methyl-group donor intake (folate, folic acid, betaine), only in the periconception period, was associated with DNA methylation in genes related to growth (*IGF2* DMR), metabolism (*RXRA*), and appetite control (*LEP*). For *LEP*, only a negative association was observed with prepregnancy folate intake. A similar negative association was found in studies from the Dutch Hunger Winter where adult men exposed to famine (low availability of methyl-group donors) *in utero* had 2.8% higher *LEP* methylation levels than their unexposed siblings [19]. In addition, we observed a negative association between *IGF2* DMR methylation and folic acid use before pregnancy and a borderline positive association in the first trimester of pregnancy. Haggarty et al. [15] observed higher levels of *IGF2* cord blood methylation when folic acid supplements were used after 12 weeks of gestation as compared to use in the periconception period. One other study reported no difference in *IGF2* DMR cord blood methylation between infants born to no, moderate, or high folic acid users before pregnancy [35]. On the other hand, Steegers-Theunissen et al. [36] reported a 4.5% higher *IGF2* DMR methylation in 17 months old children when mother used a folic acid supplement of 400 μg per day in the periconceptional period. The different reported conclusions in relation to the timing of folic acid use could be important since changes in *IGF2* methylation could have unintended consequences for health and disease. For *RXRA*, positive associations were observed with prepregnancy betaine and folate intake. Other studies have also reported both increases and decreases in methylation depending on the timing of the exposure and locus under study [19]. Ours and other results show that both hypo- and hypermethylation can be found in offspring exposed to a higher intake of methyl-group donors. There is no simple correlation between maternal methyl-group donor intake and offspring DNA methylation. It is rather believed that these epigenetic modifications in the offspring are a coordinated adaptive response to environmental challenges, whereby the developing offspring adjusts their physiology to suit the expected postnatal environment [3].

The periconception period, during which embryonic development takes place, is a vulnerable period, where nutritional exposures can disrupt the correct establishment of epigenetic marks [1]. Studies from the Dutch Hunger Winter show that adults who had been exposed to famine early in gestation and late gestation had a 5.2 and 0.9% (not significant) lower methylation of the *IGF2* DMR gene as opposed to unexposed same-sex siblings, respectively (timing specific) [2, 5]. It is thought that epigenetic marks set in the periconception period were more stable and could be maintained for many cell divisions (long-term stability), while marks set later in gestation on the other hand seem to be more flexible and can be removed within a few cell divisions (short-term flexibility) [37]. It has been suggested that the stable epigenetic marks represent those determined by genetics, whereas, the variable marks are more likely to be influenced by the environment, for example, nutrition [38].

Our results also indicate that maternal methyl-group donor intake during every trimester of pregnancy could influence *DNMT1* buccal DNA methylation in the infant. In the first and second trimester of pregnancy, lower intakes of maternal methyl-group donors resulted in higher *DNMT1* buccal DNA methylation levels. In the third trimester on the other hand, higher maternal methyl-group donor intake resulted in higher *DNMT1* methylation. Several animal studies have reported altered *DNMT1* expression in offspring due to differences in maternal diet (folic acid supplementation, low-protein diet, low-choline diet) [39–42]. Kovacheva et al. [42] reported that choline deficiency during gestation can modulate the fetal DNA methylation machinery through hypomethylation of the regulatory CpGs within the *DNMT1* gene, leading to its overexpression. It was already shown that hypomethylation of a single CpG (position 101) in the *DNMT1* gene can upregulate its expression [43], which was confirmed in the previous study. Maternal choline deficiency also resulted in an increased global and gene-specific (*IGF2*) DNA methylation in fetal livers [42]. It has already been shown that *DNMT1* is important for the maintenance of *IGF2* methylation patterns [44]. The negative associations found between *DNMT1* methylation and maternal methyl-group donor intakes are in line with the results from animal studies. This could also indicate that the observed changes in DNA methylation in the other genes could result from an increased expression of *DNMT1*. However, in the third trimester of pregnancy, a positive association was observed. This change in direction could be due to a shift in the I-C metabolism during gestation. A higher rate of transsulfuration was previously reported in the first trimester of pregnancy and a higher rate of transmethylation in the third trimester [45].

It is becoming clear that nutrition cannot only affect the epigenome in the prenatal life but that it extends into the early postnatal period. Breast milk has the ideal combination of nutrients, hormones, and other factors essential for the proper development and health of babies and seems to have a protective effect against the development of obesity later in life [23]. Exclusive breastfeeding is recommended by the World Health Organization (WHO) for the first 6 months of life [46]. In this study, we found that 6 months old infants from mothers who breast-fed for at least 3 months and had a high dietary intake of choline (≥275.27 mg/day) had statistically significant higher buccal *RXRA* CpG4 and CpG mean methylation levels. However, we were not able to reproduce these results when dividing the postnatal period in two periods (0–3 months and 3–6 months). In the second analysis, we did not find an association between maternal choline intake during lactation and buccal DNA methylation. Results from other studies show that the mean daily choline intake is about 300 mg/day [47]. These results are in line with the mean daily choline intake of women in the MANOE study (Table 2), but the choline intake is still far below the adequate intake of 450 mg/day during pregnancy and 550 mg/day during lactation. Maternal dietary choline intake can affect choline breast milk composition and infant choline status. Folate breast milk concentration on the other hand is maintained even when the mother is folate deficient and is unaffected by maternal folic acid supplementation [24]. In breast milk, choline is mainly present as phosphocholine (45%) and glycerophosphocholine (29%), with smaller amounts of free choline (9%), phosphatidylcholine (7%), and sphingomyelin (10%). Ilcol et al. [48] have demonstrated that free choline concentrations in breast milk were influenced by maternal circulating choline status (serum free choline). Also, serum-free choline concentrations in infants were correlated with free choline, phosphocholine, glycerophosphocholine, and total choline contents of breast milk.

A possible explanation for the increase in *RXRA* methylation in infants from breastfeeding mothers with higher dietary choline intake during the first 6 months could be through higher availability of methyl-group donors in the I-C metabolism. Choline is first oxidized into betaine, which contains three methyl-groups. A methyl-group is donated to homocysteine for the formation of methionine, which is further transformed to SAM. SAM will eventually donate the methyl-group to the DNA and is converted into S-adenosylhomocysteine (SAH). Thus, a higher dietary intake of choline could lead to higher DNA methylation levels through the provision of methyl-group donors [49]. Next to the role of choline in the I-C metabolism, it also plays a role in the lipid-cholesterol transport and metabolism (choline is a precursor of phosphatidylcholine) [50]. A specific species of phosphatidylcholine is the peroxisome proliferator-activated receptor alpha (*PPARα*). *PPAR* and *RXR* form heterodimers that regulate transcription of genes involved in insulin action, adipocyte differentiation, lipid metabolism, and inflammation [22]. According to a study in two independent cohorts, greater cord blood methylation of *RXRA* was strongly correlated with greater adiposity in later childhood (9 years old). A 17, 20, and 6% increase in fat mass and a 10, 12, and 4 increase in percentage fat mass were found per SD change in *RXRA* methylation [21]. A potential mechanistic pathway involved is that the induction of transcription by *RXRA* is dependent on its binding to ligands including the peroxisome proliferator-activated receptors. Our results indicate that a higher maternal choline (≥275.27 mg/day) intake during lactation was linked with higher *RXRA* methylation in infant’s buccal cells. We do not know if the positive association found between *RXRA* cord blood methylation and fat mass in 9-year-old children [21] applies for *RXRA* buccal epithelial cell methylation at 6 months of age and childhood fat mass. In this study, we were not able to link our methylation data with infant characteristics. Children of the MANOE study will therefore be followed up at a later age, to link the infant’s methylation levels with anthropometric measurements (weight, height, and fat mass).

Many studies have found an association between small DNA methylation changes at single CpGs or over a very limited genomic region (<10% and often only 1–5%) and disease phenotypes. Such small DNA methylation changes are known to be set during epigenetic sensitive periods and play a role in creating a large diversity in phenotypes linked to the onset of many complex diseases. Our study also observed small changes in DNA methylation, but the true biological relevance and how these small changes could give rise to the disease phenotype (mechanism) remains unknown [51].

There are some strengths and limitations in the present study we need to address. The strengths of the present study include a unique study design that allowed us to collect longitudinal maternal data (starting before pregnancy, during each trimester of pregnancy, and in the PP period) and offspring gene-specific DNA methylation data in buccal epithelial cells at the age of 6 months. The use of a validated food-frequency questionnaire designed to assess the intake of the nutrients under study adds to the strength of our study. In addition, at each study time point detailed information about supplement use was obtained. We also have detailed covariate data allowing for adjustment for potential confounding variables. Another advantage is the use of bisulfite pyrosequencing for DNA methylation analysis in candidate genes. It enables the determination of DNA methylation levels at individual CpG sites and the calculation of the average methylation percentage of that region. Single CpG site methylation in the promoter region of a gene can be involved in the regulation of transcription, especially when it lies in a relevant transcription factor binding site, and could be associated with diseases. From example, the loss of DNA methylation in one CpG site in the promoter region of *TET1* was associated with air pollution and childhood asthma and could possibly be a potential biomarker for childhood asthma [52]. CpG methylation within the same CpG island in promoter regions has shown to be highly correlated and these methylation patterns have been shown to differ from methylation patterns elsewhere, indicating that they have a specific biological role [53].

A first limitation is that we measured offspring methylation in buccal epithelial cells and not in the target tissue (adipose tissue). We do not know to what extent the methylation changes found in buccal epithelial cells reflect the changes in the less accessible target tissue (adipose tissue). DNA extracted from buccal swabs mainly stems from exfoliated epithelial cells but has a more homogenous cell population compared to blood samples. Buccal swabs are most often used in epidemiological studies involving young children because it is non-invasive and easy to obtain [27]. It has been shown that buccal samples are more informative than blood samples in DNA methylation studies with non-blood-based diseases/phenotypes (for example obesity) as outcome [54]. We have measured DNA methylation at the age of 6 months and we do not know if these are the methylation patterns set at birth or if it reflects methylation changes due to early postnatal exposures. Another limitation is the fact that the Belgian food composition database NUBEL [55] does not contain information about the four methyl-group donors under study. Databases of neighboring countries or the USDA database for choline and betaine [56] content were used in the validation of the FFQ [29, 30]. For folate, the Dutch NEVO food composition database was used [57] and the German BLS nutrient database for methionine [58]. The USDA database was also used for the nutrient content of folate and methionine if not found in NEVO and BSL databases, respectively. In addition, in the validation of the FFQ, we found a lower correlation coefficient (which measures the association between the FFQ and the reference method) of 0.42 for choline [29]. The correlation coefficients should be at least 0.40 and optimally in the range of 0.50–0.70 [59]. A last limitation is that we have only performed an analysis for technical variation in the pyrosequencing analysis and did not include control samples.

## Conclusions

This study suggests that maternal dietary and supplemental intake of methyl-group donors, especially in early gestation, can influence infant buccal DNA methylation in genes related to metabolism, growth, appetite regulation, and DNA methylation reactions. We have also shown that nutrition in the early postnatal period, lactation, can influence infant DNA methylation levels. Higher maternal choline intake in mothers who breast-fed their children for more than 3 months resulted in higher buccal *RXRA* methylation levels in 6 months old infants.
